# Repeat Resection for Recurrent Glioblastoma in the WHO 2021 Era: A Longitudinal Matched Case-Control Study

**DOI:** 10.3390/brainsci15050463

**Published:** 2025-04-27

**Authors:** Melike Mut, Hatice Yagmur Zengin, Aynur Azizova, Cengiz Savas Askun, David Schiff, Figen Soylemezoglu

**Affiliations:** 1 Department of Neurosurgery, University of Virginia, Charlottesville, VA 22908, USA; 2 Department of Neurosurgery, Faculty of Medicine, Hacettepe University, Ankara 06100, Turkey; 3 Department of Biostatistics, Faculty of Medicine, Hacettepe University, Ankara 06100, Turkey; 4 Radiology & Nuclear Medicine Department, Vrije Universiteit Amsterdam, 1081 HV Amsterdam, The Netherlands; 5 Cancer Center Amsterdam, Imaging and Biomarkers, 1105 AZ Amsterdam, The Netherlands; 6 Department of Radiology, Hacettepe University School of Medicine, Ankara 06100, Turkey; 7 Department of Computer Education and Instructional Technology, Middle East Technical University, Ankara 06800, Turkey; 8 Department of Neurology, Division of Neuro-Oncology, University of Virginia, Charlottesville, VA 22903, USA; 9 Department of Pathology, Faculty of Medicine, Hacettepe University, Ankara 06100, Turkey

**Keywords:** glioblastoma, IDH-wildtype, recurrent, repeat resection, surgery, survival, extent of resection

## Abstract

Background and Objectives: This study aims to evaluate the overall survival benefits of repeat resection in patients with recurrent glioblastoma, IDH-wildtype (rGBM), and to identify factors for long-term survival, including the role of clinical, radiological, and molecular parameters. Methods: This longitudinal matched case-control study included 60 patients with rGBM divided into two groups: one surgery (*n* = 30) and repeat resection (*n* = 30). The baseline characteristics, preoperative and postoperative volumes, and molecular markers were assessed. Survival analyses were conducted using the Log-rank test, and associated factors with long-term survival were identified in the repeat resection cohort. Results: The patients who underwent repeat resection had a significantly longer median survival of 23.9 months compared to 9.2 months in the one-surgery group (*p* < 0.001). Preoperative tumor volume was found to correlate with postoperative residual volume in repeat resections. The patients with no residual contrast-enhancing tumor volume (0 cm^3^) after repeat resection had a median survival of 19.33 months, while those with any residual volume had a median survival of 10.13 months. The patients with lower KPS (≤70) and GCS (≤13) scores at the time of the repeat resection tended to have shorter survival, underscoring the potential clinical relevance of functional status when evaluating surgical candidacy. Conclusions: Complete repeat resection may improve overall survival in patients with recurrent IDH-wildtype GBM and should be considered earlier as a therapeutic option rather than a diagnostic or salvage procedure. Early surgical intervention, before declines in the KPS and GCS or tumor volumes become unmanageable, may lead to better outcomes. Further studies with larger cohorts are needed to confirm these findings.

## 1. Introduction

Glioblastoma (GBM), the most aggressive primary brain tumor in adults, remains a significant therapeutic challenge. The standard of care typically involves maximal safe surgical resection followed by concurrent radiotherapy and temozolomide chemotherapy, based on the Stupp protocol [1]. Despite these intensive first-line treatments, the prognosis remains poor, with a median overall survival of 12–15 months and inevitable disease progression in most patients [2]. Once recurrence occurs, therapeutic options become limited, and no treatment has consistently demonstrated a significant improvement in survival post-recurrence. The management of recurrent GBM (rGBM) remains highly debated, with no clear consensus on the optimal approach [3].

Repeat resection is one approach that has been explored for managing rGBM, yet its efficacy remains contentious. Earlier studies often produced mixed results, partly due to heterogeneity in patient selection and molecular tumor characteristics, which were poorly understood before the 2021 WHO classification update. More recent evidence suggests that maximal safe re-resection may be associated with improved survival outcomes, particularly in patients with minimal residual tumor volume postoperatively [4,5]. Additionally, the influence of molecular profiles, such as IDH mutation status and Methylguanine-DNA methyltransferase (MGMT) promoter methylation, is becoming increasingly recognized as a critical factor influencing outcomes after reoperation [6].

Deciding whether to pursue repeat resection is a complex process that requires careful consideration of various clinical, surgical, and molecular factors. This study aims to assess the overall survival benefits of repeat resection in a well-defined cohort of patients with rGBM, as well as to identify factors that can be associated with long-term survival. To our knowledge, this is the first longitudinal matched case-control study that integrates clinical, radiological, and molecular parameters to evaluate the role of repeat resection in recurrent GBM. Furthermore, we aim to identify factors for long-term survival following repeat resection, allowing for more informed patient selection and improved treatment planning.

## 2. Materials and Methods

### 2.1. Study Design

This study utilized a longitudinal, matched case-control design involving patients diagnosed with IDH-wildtype GBM and treated between 2014 and 2019 in a single institute and the pathological specimens were re-analyzed and regraded based on the WHO 2021 molecular criteria [7]. The Institutional Review Board approved this study.

#### 2.1.1. Patient Population

We retrospectively identified patients diagnosed with glioblastoma (GBM), IDH-wildtype, who underwent initial surgery between 2014 and 2019. The tumor specimens were re-analyzed and regraded according to the 2021 WHO criteria. The patients were included if they had the following: 1. radiological and/or symptomatic tumor progression following initial treatment; 2. tumors deemed surgically accessible for repeat resection; 3. failure of non-surgical therapies or requirement of tissue sampling for further treatment planning; 4. longitudinal follow-up until death; and 5. an American Society of Anesthesiologists (ASA) score between 1 and 3 (indicating fitness for surgery) [8]. The patients were required to meet all these criteria. The patients with multicentric or multifocal GBMs requiring multiple craniotomies at first presentation and severe comorbidities precluding safe anesthesia at initial presentation were excluded. Contraindications for repeat resection included inaccessible tumor locations, extensive disease spread, or severe comorbidities that had a significant impact on the patient’s surgical procedure. The decision-making process involved a multidisciplinary team to thoroughly evaluate and balance the potential benefits and risks.

#### 2.1.2. Group Allocation and Matching

A total of 136 patients with recurrent GBM were evaluated for repeat surgery. Of these, 102 met the inclusion criteria and enrolled in this study; 32 underwent repeat resection, while 70 declined surgery. An additional 34 patients underwent repeat resection during the same period but were excluded for failing at least one inclusion criterion. From the 32 patients who had repeat surgery, 2 were excluded because of unsatisfactory MRI findings and a lack of viable tumor on pathology (consistent with radiation necrosis), leaving 30 patients in the repeat resection group. The remaining 30 patients met the eligibility criteria with confirmed recurrent disease in the pathological examination of the specimens. Each of these 30 was matched one-to-one to a patient who refused a second surgery (*n* = 30), creating two final cohorts:

- One-surgery cohort (*n* = 30).
- Repeat resection cohort (*n* = 30).

**Figure 1:**
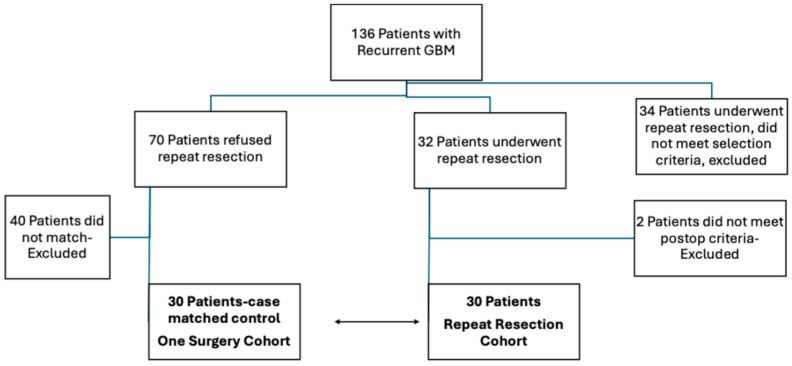
Flowchart of patient selection. The arrow shows matched cohorts.

The patients were matched by age, sex, preoperative tumor volume at initial diagnosis, comorbidities (hypertension and diabetes), ASA score, extent of resection (EOR) at initial surgery, tumor location (left vs. right hemisphere; eloquent vs. non-eloquent areas), Glasgow Coma Scale (GCS), Karnofsky Performance Scale (KPS), postoperative adjuvant therapies, and selected molecular markers (MGMT methylation, TERT mutation, p53, and ATRX loss). Forty patients could not be matched and were excluded (Figure 1).

#### 2.1.3. Study Flow and Interventions

##### Initial Surgery and Follow-Up

All the initial resections were performed by the senior author (M.M.). The extent of resection was determined postoperatively via MRI (≤48 h after surgery). After standard first-line therapy (Stupp protocol), the patients were followed longitudinally until death, receiving additional lines of treatment (e.g., second- or third-line chemotherapy, reirradiation, or immunotherapy) as needed.

##### Offering Repeat Resection

The patients meeting eligibility were offered a second surgery. Some declined for personal or logistical reasons. The reasons for declining surgery included concerns about potential risks or complications, a lack of belief in the surgical benefits, a preference to pursue second- or third-line medical treatments, family decisions, long travel distances for follow-up, and an inability or unwillingness to adhere to the close follow-up requirements of this study. Among those who underwent repeat resection, the procedure was again performed by the same surgeon (M.M.), and the postoperative imaging protocol was repeated.

#### 2.1.4. Analysis

Volumetric analyses of contrast-enhancing (CE) tumors were performed using a semi-automated method. In short, the tumor volumes were quantified on pre- and postoperative MRI scans (obtained ≤48 h after surgery). The total contrast-enhancing (CE) tumor volume was quantified using a semi-automated volumetric segmentation method in 3D Slicer (version 5.6.2) on contrast-enhanced T1-weighted MRI sequences, and non-contrast-enhancing (nCE) tumor was delineated on FLAIR or T2 sequences. The absolute volumes (cm^3^) were recorded for both first and repeat resections and the relative volume reduction was calculated as (postoperative volume/preoperative volume) × 100. The Response Assessment in Neuro-Oncology (RANO) classification system for GBM divides the EOR into four primary categories based on residual tumor volume. Class 1 signifies supramaximal resection, which is defined as 0 cm^3^ of CE tumor and ≤5 cm^3^ of nCE tumor [5,9,10]. For patients with no postoperative residual CE tumor, the MRIs were further analyzed in terms of the volume of the nCE tumor to determine their RANO scales.

#### 2.1.5. Data Collection and Variables

##### Clinical Variables

We recorded age, sex, hypertension, diabetes mellitus (DM), ASA score, GCS, and KPS at presentation and at the time when the repeat resection was offered. The time to first progression was assessed per the RANO 2.0 criteria [5,9,10].

##### Molecular Markers

The specimens were evaluated for IDH status, MGMT promoter methylation, TERT mutation, p53, and ATRX loss, following the 2021 WHO classification guidelines.

##### Outcome Measures

Overall survival was calculated from the date of initial surgery to time of death. Eloquence was defined as the involvement of motor, sensory, and speech areas, and these factors were matched one-to-one, as was the left-side localization of the tumors. The time to first progression was assessed per the RANO 2.0 criteria [10]. We defined “time to first progression” as the interval from the date of the initial surgery to the initial radiological or clinical detection of recurrence. In contrast, “time to offer repeat resection” corresponds to the point at which the repeat surgery was both feasible and accepted by the patient. Additional analyses focused on the impact of functional status (KPS and GCS) on survival after repeat resection. In the repeat resection group, we further compared “short-term” vs. “long-term” survivors (*n* = 7 vs. *n* = 23) to explore potential clinical or molecular predictors of extended survival.

#### 2.1.6. Statistical Analysis

The aim of this analysis was to assess whether patients undergoing repeat resection for recurrent glioblastoma (rGBM) had significantly different baseline characteristics compared to those undergoing a single surgery. A 1:1 matched case-control design was used, controlling for key variables such as age and sex and initial tumor characteristics.

The analysis was conducted using IBM SPSS Statistics version 23 for Windows, JASP, and R Studio Version 1.4.1106 with R version 4.0.4. The R Packages used for this study were factoMineR, factoextra, haven, survival, survminer, and ggplot2. We have included additional visualizations and detailed data plots in the Supplementary Material (Supplementary File), allowing for a clearer depiction of the demographic and clinical variables.

Multivariate outliers in terms of baseline clinical and demographic characteristics were examined using the Factor Analysis of Mixed Data (FAMD) method, and graphical representations are presented as scatter plots in the Supplementary File. The identified outliers were not excluded in the explanatory analysis due to their clinical relevance. Missing data were handled using a pairwise deletion approach in the univariate analyses, and no imputation method was applied, as the missing values occurred only in the categorical variables.

The numerical variables used in this study were visualized using raincloud plots with jittering to enhance interpretability (Supplementary File). The normality of numerical variables was assessed using the Shapiro–Wilk test for subgroups with sample sizes < 50 (e.g., surgery and survival groups) and the Kolmogorov–Smirnov test for larger samples (*n* > 50). Homogeneity of variances was evaluated using Levene’s test. When assumptions for parametric tests (i.e., normality and/or homogeneity of variances) were violated, the Mann–Whitney U test was used for independent group (e.g., surgery and survival groups) comparisons of the numerical data. The categorical variables were analyzed using Pearson’s Chi-square test or Fisher’s Exact test, as appropriate. Hypothesis testing was conducted with a significance threshold of α = 0.05. Cohen’s d (for numerical variables) and Phi coefficients (for categorical variables) were calculated to assess the effect sizes and presented with a 95% confidence interval (95% CI).

Overall survival (OS) was evaluated using the Kaplan–Meier survival analysis, and the survival curves between the surgery groups were compared using the Log-rank test.

A simple linear regression analysis was used to explore the relationship between the preoperative and postoperative tumor volumes at repeat resection. Assumptions of linear regression were assessed: the normality of residuals was tested using the Shapiro–Wilk test. Influence, distance, and residual statistics were evaluated to identify influential, distant, and outlier observations. Two observations (see Supplementary File) were identified as influential outliers, contributing to non-normally distributed residuals. Homoscedasticity was assessed visually (residual vs. predicted value scatter plot) and statistically using the Breusch–Pagan test. Autocorrelation was evaluated using the Durbin–Watson test. Since assumptions were not met, a log(y + 1) transformation was applied to the dependent variable (postoperative tumor volume), and the two influential outliers were excluded from the analysis.

Similarly, the relationship between postoperative volume and survival after repeat resection was analyzed using simple linear regression, and all the regression assumptions were tested.

All the statistical tests were two-tailed, and a *p*-value < 0.05 was considered statistically significant.

## 3. Results

### 3.1. Baseline Characteristics

**Figure 2:**
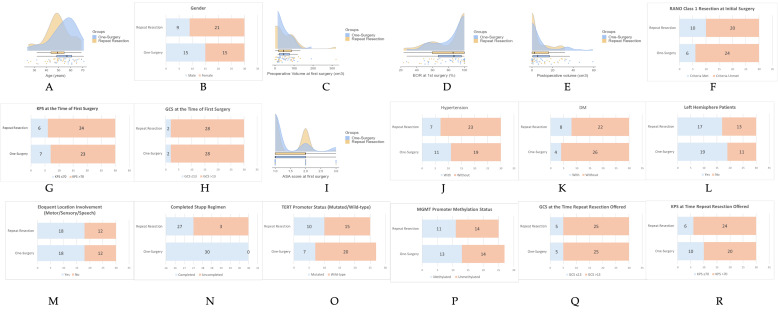
Distribution of demographic, clinical, and surgical variables in patients undergoing single surgery versus repeat resection. Panels (**A**,**C**–**E**,**I**) show raincloud plots for continuous variables: (**A**): age, (**C**): preoperative volume, (**D**): extent of resection (EOR) at first surgery, (**E**): postoperative volume, and (**I**): ASA score at first surgery. Panels (**B**,**F**–**R**) display bar plots for categorical variables including the following: (**B**): gender, (**F**): RANO Class 1 resection, (**G**): KPS at the time of first surgery (≤70/>70), (**H**): GCS at the time of first surgery (≤13/>13), (**J**): hypertension, (**K**): diabetes mellitus (DM), (**L**): left hemisphere involvement, (**M**): eloquent cortex involvement, (**N**): Stupp regimen completion, (**O**): TERT mutation status, (**P**): MGMT methylation, (**Q**): GCS at time of repeat resection, and (**R**): KPS at time of repeat resection.

The median age of the patients undergoing one surgery was 54.1 (11.05) years (range: 24–71), compared to 49.3 (10.74) years (range: 25–69) for those undergoing repeat resection (Student’s *t*-test t = 1.706; df = 58; and *p* = 0.093). The distribution of the preoperative tumor volumes was similar between the groups. The median preoperative volumes of the one-surgery and repeat resection groups were 46.1 cm^3^ and 45.5 cm^3^, respectively (Mann–Whitney U z(60) = −0.163; *p* = 0.871). The gender distribution was also statistically similar in the one-surgery vs. repeat resection groups (Pearson χ^2^ = 2.5, df = 1, and *p* = 0.114; 15 females and 15 males vs. 21 females and 9 males, respectively). The distribution of the comorbidities such as hypertension (Pearson χ^2^ = 1.27; df = 1; and *p* = 0.260) and diabetes mellitus (DM) (Pearson χ^2^ = 1.667; df = 1; and *p* = 0.197) showed no significant differences between the one-surgery and repeat resection groups. The median of The American Society of Anesthesiologists (ASA) score at first surgery was 1 in the one-surgery and 2 in the repeat resection groups (Mann–Whitney U z(60) = 0.778; *p* = 0.437). There was no difference in their KPS and GCS at initial presentation, statistically (Table 1). Ten patients in the one-surgery and eleven patients in the repeat resection group received second-line chemotherapy before the time they were offered repeat resection and they included bevacizumab, irinotecan, lomustine, or carboplatin. No significant differences in the second-line medical therapies were observed between the patients with one surgery and with repeat resection (Pearson χ^2^ = 0.073; df = 1; and *p* = 0.787). The median time to offer re-resection was 7.17 months (range: 1.9–14.5 months) for the one-surgery group and 11.63 months (range: 3.8–57.1 months) for the repeat resection group, with a statistically significant difference (Mann–Whitney U z(60) = 2.706; *p* = 0.007). In the repeat resection group, once the surgical option was presented and accepted, the procedures were performed promptly thereafter. At the time re-resection was offered, 5 patients in each group had a GCS score of ≤13 (Pearson χ^2^ = 0; df = 1; and *p* = 1), while 10 patients in the one-surgery group and 6 patients in the repeat resection group had a KPS score of ≤70 (Pearson χ^2^ = 1.364; df = 1; and *p* = 0.243) (Table 1 and Figure 2). The reasons for refusing surgery in the one-surgery group were as follows: concerns about potential risks or complications (*n* = 9), disbelief in the surgical benefits (*n* = 10), a preference for second- or third-line medical treatments (*n* = 11), family decisions (*n* = 5), long travel distances for follow-up (*n* = 8), and an inability or unwillingness to comply with this study’s intensive follow-up requirements (*n* = 3). The 26 of 30 patients who underwent only one surgery continued with second- or third-line chemotherapy, and 1 patient additionally received repeat radiotherapy with nivolumab.

We excluded the 40 patients who underwent one surgery as that was not within the scope of this manuscript due to the one-to-one case-control matching design with the strict matching criteria; however, it is worth mentioning that among the excluded patients who underwent only one surgery, the median age was 68 years, with 23 males and 17 females. The median overall survival in this group was 10.1 months.

### 3.2. EOR at Initial Surgery and Molecular Markers

The EOR during the initial surgery was similar between the groups, with 86.9% for the one-surgery group and 85.8% for the repeat resection group (Mann–Whitney U z(60) = −0.104; *p* = 0.917). The median residual tumor volume at the first surgery was 5.61 cm^3^ (range: 0–59.09) for the one-resection group and 2.38 cm^3^ (range: 0–31.99) for the repeat resection group (Mann–Whitney U z(60) = −0.940; *p* = 0.347). RANO Class 1 resection during the first surgery was achieved in 6 patients in the one-surgery group and 10 patients in the repeat resection group (Pearson χ^2^ = 1.364; df = 1; and *p* = 0.243). The tumor locations—whether in the left hemisphere or in eloquent (motor, sensory, and speech) regions—did not differ significantly between the groups (Pearson χ^2^ = 0.278; df = 1; and *p* = 0.598 and Pearson χ^2^ = 0; df = 1; and *p* = 1, respectively). Adverse effects from the initial surgery were noted in five patients in the repeat resection group, including temporary dysphasia in two patients and permanent visual field deficits in three patients. In the one-surgery group, six patients experienced complications, comprising temporary dysphasia in two patients, temporary motor dysfunction in two patients, and permanent visual field deficits in two patients. MGMT methylation and TERT promoter mutation status were not significantly different between these groups (Pearson χ^2^ = 0.09; df = 1; and *p* = 0.764 and Pearson χ^2^ = 1.169; df = 1; and *p* = 0.280, respectively) (Table 1). The cohort in this study was composed entirely of IDH-wildtype tumors and the histone H3 status was not assessed due to the absence of radiological or pathological indications for midline glioma. Other molecular markers like CDKN2A/B, KDR, and EGFR were not available for all the patients at the time of study.

### 3.3. Overall Survival

**Figure 3:**
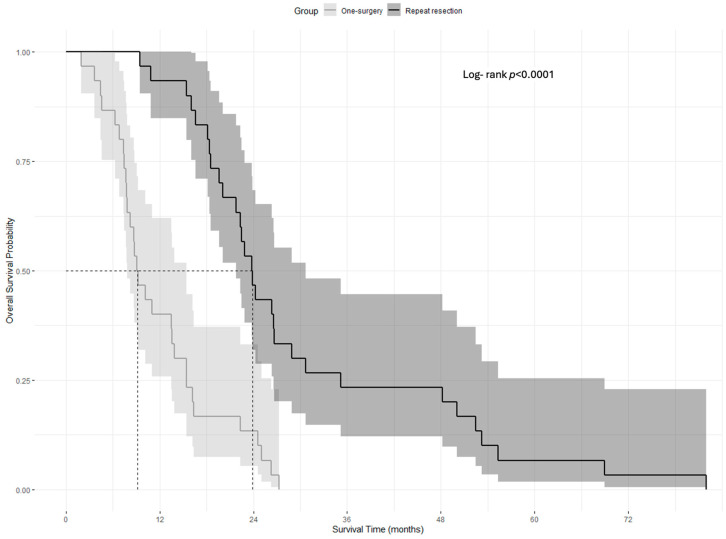
Kaplan–Meier analysis of overall survival between one-surgery and repeat resection cohorts with 95% confidence intervals.

All the patients in this cohort died during the study period. The median overall survival was 9.17 months (95% CI: 7.87–15.4 with range: 1.93–27.3 months) for the one-surgery group and 23.87 months (95% CI: 21.0–30.7 with range: 9.47–81.97 months) for the repeat resection group, demonstrating a statistically significant difference (Mann–Whitney U z(60) = 5.108; *p* < 0.001) (Table 2). The Log-rank test following the Kaplan–Meier analysis indicated that the one-surgery versus repeat resection groups were statistically different in terms of OS distribution (Log-rank test χ^2^ = 28.501, df = 1, and *p* < 0.001; Figure 3).

The survival time after repeat resection was offered was 2 months (95% CI: 1.857–2.143 with range: 0.033–14.267 months) in the one-surgery group compared to 11.35 months (95% CI: 9.121–12.879 with range: 1.967–50.57 months) in the repeat resection group (Mann–Whitney U, z(60) = 4.348; *p* < 0.001) and it was statistically significant. The Log-rank test indicates that the one-surgery versus repeat resection groups were statistically different in terms of survival time distribution after the repeat resection was offered (Log-rank test statistic = 21.106 with df = 1; *p* < 0.001). In the repeat resection group, the median extent of resection (EOR) was 88.17% (range, 10.73–100), with a median residual tumor volume of 3.27 cm^3^ (range: 0–94.84). According to the RANO Class 1 criteria, 8 out of 22 patients met the standard for complete resection (Table 2).

### 3.4. Factors Related to Long Survival After Repeat Resection

All the patients after the repeat resection had a better GCS and their KPS remained stable without additional neurological deficits. In the repeat resection cohort, 7 out of 23 patients did not survive long enough to receive postoperative adjuvant therapy. The remaining 16 patients received second/third-line chemotherapies, such as temozolomide, irinotecan, bevacizumab, and etoposide/cisplatin. Four long-term survivors after repeat resection received repeat radiotherapy with nivolumab. In the short-term survivors of the repeat resection group, the median survival after repeat resection was 2.1 months (range: 1.97–3.50) compared to 12.77 months (range: 6.07–50.57) in the patients who were able to receive adjuvant therapy and achieved longer survival. We compared the patients who had short survival (*n* = 7) to those who had long survival (*n* = 23) after the repeat resection to identify associated factors for long-term survival (Table 3). Among several factors, only the preoperative GCS (Fisher’s Exact test, *p* < 0.001) and preoperative KPS (Fisher’s Exact test, *p* = 0.007) were significantly different between the short-term and long-term survivors following repeat resection. The Park score, which combines the preoperative tumor volume, KPS, and tumor location within the motor, speech, or middle cerebral artery territory, was influential but did not achieve statistical significance, likely due to the limited sample size (Mann–Whitney U, z(30) = −1.274; *p* = 0.203). Among the surgical markers, eloquent (motor, sensory, and speech) location involvement was observed in 3 of the short-term survivor group vs. 15 of the long-term survivor group (Fisher’s Exact test, *p* = 0.392) indicating no statistically significant difference. Similarly, 4 vs. 13 patients had left-hemisphere tumors (Fisher’s Exact test, *p* = 1), contact with the ventricle wall was documented in 6 vs. 16 patients (Fisher’s Exact test, *p* = 0.638), and ependymal enhancement occurred in 2 vs. 11 patients, respectively, in the short-term survivor and the long-term survivor groups (Fisher’s Exact test, *p* = 0.427). Overall, none of these surgical markers reached statistical significance between the two groups (Table 3).

**Figure 4:**
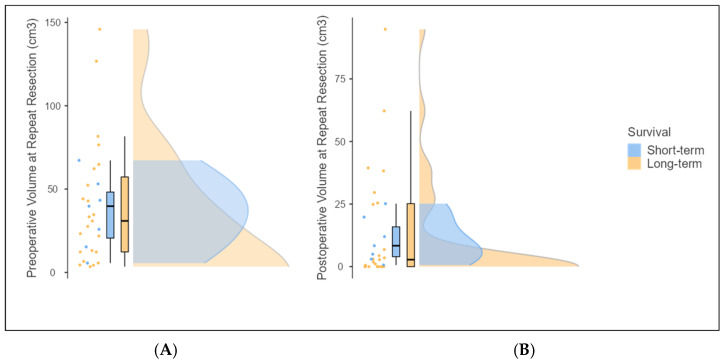
Distribution of preoperative (**A**) and postoperative (**B**) volumes in short-term and long-term survivors in repeat resections.

The preoperative volume at repeat resection was 39.76 cm^3^ (range: 5.61–67.24) in the short-term survivor group and 30.89 cm^3^ (range: 3.47–145.9) in the long-term survivor group, with no significant difference observed (Mann–Whitney U, z(30) = −0.270; *p* = 0.811). In comparing the short-term and the long-term survivor subgroups, the postoperative tumor volume at repeat resection was 8.35 cm^3^ (range, 0.62–25.16) for the short-term survivors and 2.79 cm^3^ (range, 0–94.84) for the long-term survivors (Mann–Whitney U, z(30) = −1.015, *p* = 0.335, and d = 0.19 [95% CI: −1.035 to 0.660]) (Figure 4A,B). Similarly, the extent of resection (EOR) at repeat resection was 78.99% (range, 41.88–90.64) in the short-term survivors versus 89.45% (range, 10.73–100) in the long-term survivors (Mann–Whitney U, z(30) = 1.163, *p* = 0.287, and d = 0.07 [95% CI: −0.915 to 0.777]) (Table 3). However, the long-term survivors have more variability in pre- and postoperative volumes compared to the short-term survivors (Figure 4A,B).

**Figure 5:**
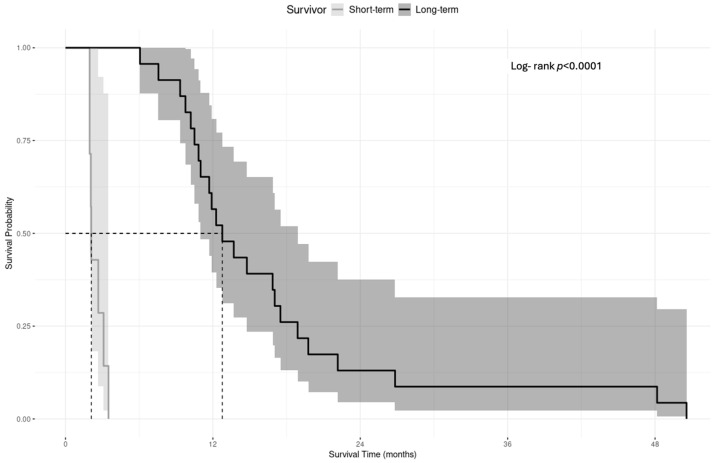
Kaplan–Meier analysis of survival after repeat resection between short-term and long-term survivor cohorts with 95% confidence intervals.

The Log-rank test reveals a statistically significant difference in survival after repeat resection between the short- and long-term survivor groups (Log-rank *p* < 0.001; Figure 5). This suggests that the survival distributions of the two groups are significantly different over time.

**Figure 6:**
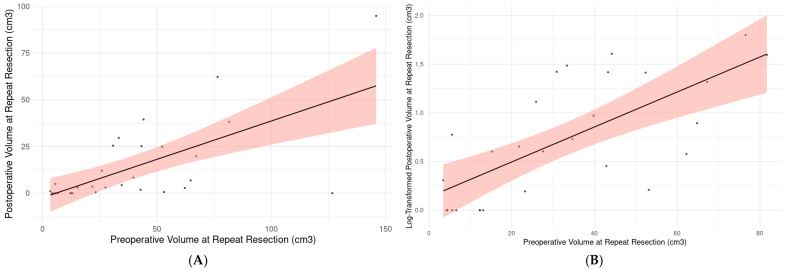
(**A**): Relationship of preoperative and postoperative volume in repeat resection for rGBM patients (linear regression equation: Postoperative Volume at Repeat Resection = −2.393 + 0.410 × Preoperative Volume at Repeat Resection, R-square: 0.433; *p* < 0.001). (**B**): Relationship of preoperative and log-transformed postoperative volume in repeat resection for rGBM patients while two influential and outlier patients were excluded (linear regression equation: log (Postoperative Volume at Repeat Resection) = 0.136 + 0.018 × Preoperative Volume at Repeat Resection, R-square: 0.494; *p* < 0.001). All regression lines are presented with 95% prediction bands represented with red area.

The relationship between preoperative and postoperative volumes in repeat resection was analyzed using a linear regression model and it was found that higher preoperative volumes before the repeat resection were associated with higher postoperative volumes (Table 4) (Figure 6A,B). The preoperative volumes may moderately be associated with postoperative residuals after repeat resection with a positive linear relationship, as demonstrated by the statistically significant results (R-square: 0.433; *p* < 0.001) (Table 4).

**Figure 7:**
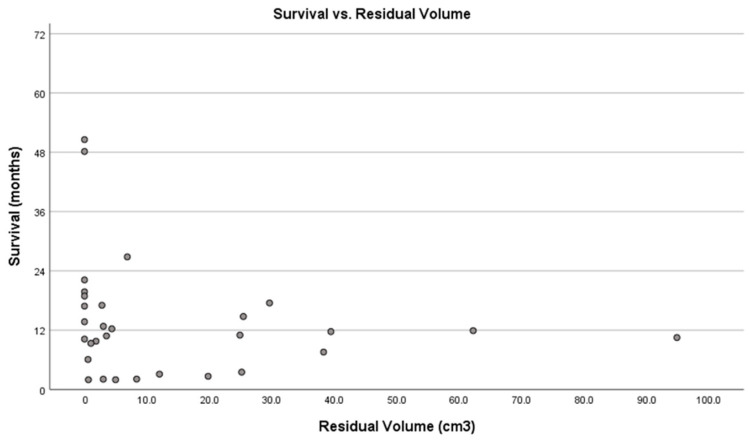
Scatter plot of residual volume after repeat resection versus survival of rGBM patients. (linear regression equation: Residual Volume = 14.931 − 0.098 × Postoperative Volume at Repeat Resection, R square = −0.033; *p* = 0.334). Dots represent the postoperative residual volume at repeat resection (cm^3^) vs. survival time after repeat resection (months).

The residual tumor volume and extent of resection did not reach statistical significance following repeat surgery. Although smaller postoperative volumes appeared to be associated with prolonged survival, the difference was not statistically significant (Mann–Whitney U z(30) = −1.015; *p* = 0.322 and z(30) = 1.163; *p* = 0.287, respectively). The residual volume after repeat resection was notably smaller among the long-term survivors (long-term survivors = 2.79 (0–94.84) cm^3^ vs. the short-term survivors = 8.35 (0.62–25.16) cm^3^). The linear regression analysis showed a weak relationship (Table 5), which suggests a more noticeable trend that higher residual volumes are linked to shorter survival (R square = 0.033; *p* = 0.334) (Table 5; Figure 7). Transformations and excluding influential outliers did not improve the regression model’s fit. Additionally, several non-linear models were also applied, yet none of them has a high R-squared value. The initial simple linear regression model results are shown in Table 5. RANO Class 1 resection was attained in 8 out of 23 long-term survivors but 0 out of 7 short-term survivors. However, the difference was not significant, probably due to the sample size (Fisher’s Exact test, *p* = 0.143).

To determine if there was a strong cutoff point in the data, we further analyzed whether a specific threshold of residual volume could significantly separate survival outcomes. The difference occurs at the 0.0 cm^3^ threshold. The patients with no residual volume have a median survival after repeat resection of 19.33 (10.20–50.57) months, while those with any residual volume above 0.0 cm^3^ had a median survival after repeat resection of 10.13 (1.97–26.83) months after the repeat resection and this was statistically significant (Mann–Whitney U, z(30) = −3.143; *p* = 0.002). This suggests that complete resection with 0.0 cm^3^ residual volume might be associated with better survival outcomes after repeat resection (Figure 7).

## 4. Discussion

Our study demonstrates a significant overall survival benefit for the patients undergoing repeat resection in recurrent GBM, with a median survival of 23.87 (9.47–81.97) months, longer than the 9.17 (1.93–27.3) months observed in the patients with only one surgery (Table 2). These findings support the earlier consideration of repeat resection, particularly before consciousness (GCS) and functional deterioration (KPS) occur. Furthermore, smaller preoperative tumor volumes were associated with less residual tumor after surgery. Notably, the patients with no residual tumor volume after repeat resection had a median survival of 19.33 (10.20–50.57) months, compared to 10.13 (1.97–26.83) months for those with residual tumors, suggesting that RANO Class 1 resection may lead to better survival outcomes.

GBM is an extremely aggressive brain tumor that inevitably recurs, even after intensive first-line treatment [2,12]. Currently, therapeutic strategies for recurrent glioblastoma are inadequate, as no treatment has demonstrated significant improvement in post-recurrence survival in randomized controlled trials [3,13]. While earlier studies have reported mixed outcomes with surgical resection at tumor progression, recent evidence suggests that repeat resection may be associated with improved survival outcomes [4,11,14,15]. Moreover, patients selected for repeat resection often have a more favorable clinical profile and less extensive, non-eloquent disease, raising questions about whether the observed benefits are due to selection bias or the EOR itself [16,17]. There is a notable literature gap regarding the selection process for patients with GBM, which presents inherent challenges for conducting prospective controlled trials. These challenges arise from various factors, including ethical considerations, tumor location, patient comorbidities, surgeons’ approaches, and molecular subgroups of GBM. To address these issues, we designed a study with matched case-control design to exclude confounding factors.

While some studies have not shown favorable outcomes with surgical resection at tumor progression, recent evidence suggests that repeat resection may be associated with improved outcomes [4,11,14,15]. Our study demonstrated the benefits of repeat resection in rGBM through a matched case-control, longitudinal approach. Repeat surgery provides patients with “a fresh start”, effectively resetting the clock to the initial time point, offering survival outcomes comparable to those seen after the first resection (11.63 vs. 11.35 months, time to second surgery vs. survival after second surgery, respectively). While repeat resection offers a valuable cytoreductive strategy in recurrent GBM, the potential for survival prolongation is likely maximized when coupled with continued chemotherapy, immunotherapy, or repeat radiotherapy. This highlights the necessity of integrating surgical intervention within a comprehensive treatment paradigm that prioritizes sustained chemotherapy delivery to fully leverage potential survival advantages. Our findings show that patients in the repeat resection group had significantly longer intervals before repeat resection was offered compared to those who only underwent one surgery. This reflects the clinical reality that repeat resection is often not the immediate next step upon first progression. Many patients remain stable long enough to attempt multiple treatment lines or display more indolent disease progression—factors that can increase the proportion of long-term survivors within the repeat resection cohort. Clarifying this timeline distinction underscores the importance of distinguishing between “time to first progression” and “time to offer repeat resection” when interpreting survival outcomes. Future research should further explore the synergistic interaction between repeat resection and specific adjuvant therapy regimens in optimizing survival outcomes.

Recent studies have emphasized the importance of minimizing residual tumor volume during reoperation to maximize patient survival, suggesting that the surgical goal should be maximal safe resection for eligible patients [6,8,18,19]. Patients selected for repeat resection often have a more favorable clinical profile and less extensive, non-eloquent disease, which raises the question of whether the observed benefits are truly due to the EOR or simply a result of selection bias [17]. The EOR has consistently been identified as a key prognostic factor, with studies highlighting that minimizing residual tumor volume during both initial and repeat surgeries is crucial for improving survival. Comparing previous surgical studies is complicated by inconsistent terminology used to describe the EOR, often referring to relative tumor reduction in percentage terms. The study by Yong et al. analyzed the impact of residual tumor volume on the survival of patients with rGBM who underwent reoperation. The median postoperative survival was 12.4 months, and results indicated that larger residual tumors (>3 cm^3^) were associated with significantly decreased survival compared to smaller or no residual tumors. The findings emphasize the importance of minimizing residual tumor volume during reoperation to maximize patient survival [18]. Given that absolute residual tumor volume (in cm^3^) might be more prognostically significant than relative resection extent, the “RANO classification for EOR” was recently established based on residual CE and nCE tumors to standardize terminology [19]. This classification aims to provide a more consistent and prognostically relevant measure of surgical success, thereby improving the design of future clinical trials and patient management strategies [5,20]. A recent study by the RANO resect group investigated the impact of repeat resection on the outcomes of patients with recurrent IDH-wildtype GBM. Data from 681 glioblastoma patients with first tumor recurrence were collected, showing that all tumors were IDH-wildtype GBM WHO grade 4. Re-resection was performed in 310 patients (45.5%), while 371 patients (54.5%) were managed non-surgically. The study found no distinct molecular profile for tumors selected for repeat resection, but these tumors often had a superficial location. Patients who underwent repeat resection typically had “supramaximal” or “maximal” resection of a CE tumor initially and were generally younger with a higher KPS at recurrence. Re-resection was associated with favorable outcomes, with a median overall survival of 11 months after recurrence compared to 7 months for non-surgically managed patients. This survival benefit persisted after adjusting for clinical confounders. The study found that only patients with residual CE tumor volumes ≤ 1 cm^3^ had improved outcomes, and the RANO classification system effectively stratified patients [9]. Preoperative tumor volume has been identified as a significant factor for survival in rGBM previously [11]. In our study, we demonstrated for the first time that larger preoperative volumes tend to be associated with larger postoperative residual volumes; for every 1 mL increase in preoperative volume, the postoperative volume increased by approximately 0.43 mL after repeat resections. This finding underscores the impact of initial tumor size on surgical outcomes in rGBM and supports the need for early surgical intervention in managing the disease.

Repeat surgery related series in favor of repeat resections [4,11,14,15] were mainly based on molecularly ill-defined cohorts prior to the 2021 WHO classification [21]. A recent study by Dono et al. analyzed data from 273 patients treated for rGBM, finding that genetic factors like CDKN2A/B loss and KDR mutations were linked to shorter post-progression survival. Repeat surgery, especially when combined with bevacizumab, improved post-progression survival, particularly in younger patients with better performance status, extending survival by 7.0 months in IDH-wildtype rGBMs. Patients with specific genetic profiles, such as EGFR mutations and CDKN2A/B mutations, benefited most from repeat resection. The study suggests that while maximal safe re-resection improves survival overall, certain genetic profiles gain greater benefit [6]. In our series, we did not identify any specific genetic feature linked to improved survival.

Level of consciousness and functional status at the time of repeat resection are critically important factors in determining outcomes. Several studies emphasize the critical role of the KPS in the prognosis of patients with IDH-wildtype GBM. Chen et al. demonstrated that a higher KPS, along with supramaximal resections, significantly improves survival [22]. Similarly, Park et al. showed that repeat resection for rGBM led to better survival outcomes, with a higher post-treatment KPS strongly associated with improved functional status and longer survival [23]. Our findings are consistent with previous studies, showing that a KPS ≤ 70 is significantly associated with short-term survival. Our study specifically emphasizes the importance of performing surgery while patients still maintain stable or only slightly deteriorated functional status (KPS/GCS), thus offering a complementary perspective to large-scale studies focused on timing. Our study is the first to incorporate the GCS into prognostication for repeat surgery in rGBM, revealing that patients with a GCS score of equal or less than 13 are directly linked to shorter survival [24].

We acknowledge that selection bias remains the primary limitation of our single-center case-control design and small patient cohorts. To mitigate this, we implemented a rigorous approach, incorporating strict case matching based on a comprehensive set of demographic, clinical, molecular, and radiologic criteria. Furthermore, this study is strengthened by detailed longitudinal follow-up data, although residual bias may still limit the generalizability of the findings. The repeat resection cohort comprises young and fit patients who may not be representative of the typical GBM population. The significant difference in the median time to offer re-resection (7.17 months vs. 11.63 months; *p* = 0.002) suggests a potential selection bias, where patients in the one-surgery group may have had more aggressive tumor biology, resulting in faster disease progression and a limited window for repeat resection. This shorter interval implies that tumor behavior and clinical status at recurrence influenced surgical eligibility, while patients in the repeat resection group likely had a more indolent disease course and better functional status, allowing for a longer interval before re-resection. Although not statistically significant, a higher proportion of patients in the repeat resection group had a higher KPS at the time of surgery, further indicating that clinical stability and tumor biology played a role in surgical decision-making. These factors likely contributed to the observed survival differences between the groups. At our institution, a KPS ≤ 70% or GCS ≤ 13 is not considered an absolute contraindication for surgery if the multidisciplinary team believes that resection can offer potential benefit (e.g., cytoreduction, symptom relief, and tissue diagnosis for treatment planning) and the patient is medically stable enough to undergo the procedure. This is particularly relevant in recurrent GBM where non-surgical options are limited, and even patients with some functional decline may benefit from carefully considered surgical intervention. While better functional status is generally preferred, our institutional practice does not automatically exclude patients based solely on these scores if potential benefit is perceived. Although our sample size is limited compared to larger multicenter cohorts (e.g., the RANO resect group), our study offers a valuable clinical insight regarding the optimal timing of repeat surgery. Several clinical variables, including hypertension status, GCS, and KPS scores, were reported in binarized form using clinically relevant thresholds. This approach, while practical for small-sample comparisons, limits the ability to assess continuous variability and precludes the use of certain statistical techniques, such as repeated-measures ANOVA. Early surgical intervention may offer meaningful survival benefits, especially when patients have adequate cognitive reserve and performance status. To further validate our results, future research should consider prospective study designs or recruit larger and more diverse patient populations. The considerable proportion of patients who refused repeat resection despite meeting eligibility criteria emphasizes a significant challenge in translating potential surgical benefits into real-world application for patients with recurrent GBM. Patient decisions to decline surgery, driven by factors such as risk aversion, skepticism regarding benefit, or preference for alternative treatments, reveals the complex landscape of patient perspectives in recurrent GBM. This highlights the significant challenges beyond clinical eligibility in effectively offering and implementing surgical interventions for this patient population. A clinical trial with an expanded cohort would be instrumental to reaffirm the borderline significance observed in key parameters, including the Park score, RANO criteria, and the relationship between preoperative and postoperative tumor volume. Such a trial would have enhanced impact if conducted through multi-institutional collaborations.

## 5. Conclusions

Complete repeat resection appears to improve overall survival in patients with recurrent GBM, IDH-wildtype, and may have therapeutic value beyond its traditional role as a diagnostic or salvage procedure. Early surgical intervention aimed at achieving complete resection should be considered to potentially optimize outcomes before patients develop a low KPS, a low GCS, or tumor volumes that exceed safe surgical thresholds. However, while our study found a notable survival difference at the 0.0 cm^3^ threshold for contrast-enhancing (CE) residual tumor, RANO Class 1 resection did not reach statistical significance. This underscores the need for caution in interpreting these results. Further research with larger, multi-institutional cohorts is necessary to confirm these findings for patients with recurrent GBM.

## Figures and Tables

**Table 1:** Baseline characteristics of patients in the one-surgery and repeat resection groups, matched using a case-control design (* Student’s *t*-test, ^#^ Mann–Whitney U test, ^$^ Pearson χ^2^ test, and ^π^ Fisher’s Exact test; *p* < 0.05).

	One Surgery (*n* = 30)	Repeat Resection (*n* = 30)	Test Statistics; *p*	Effect Size (Cohen’s d/Phi with 95% CI)
Age (years)	54.1 (11.05)	49.3 (10.74)	t = 1.706; df = 58; *p* = 0.093 *	d = 0.44 (−0.074–0.951)
Female/Male	15/15	21/9	χ^2^ = 2.5; df = 1; *p* = 0.114 ^$^	Phi = −0.20 (−0.451–0.045)
Preoperative Volume at First Surgery (cm^3^)	46.1 (14.1–191.3)	45.5 (1–320.4)	z(60) = −0.163; *p* = 0.871 ^#^	d = −0.11 (−0.613–0.400)
EOR at Initial Surgery (%)	86.9 (27.4–100)	85.8 (23.6–100)	z(60) = −0.104; *p* = 0.917 ^#^	d = 0.12 (−0.384–0.629)
Postoperative Volume (cm^3^)	5.61 (0–59.1)	2.38 (0–32)	z(60) = −0.940; *p* = 0.347 ^#^	d = 0.25 (−0.263–0.753)
RANO Class 1 Resection at Initial Surgery (Criteria Met/Unmet)	6/24	10/20	χ^2^ = 1.364; df = 1; *p* = 0.243 ^$^	Phi = 0.15 (−0.115–0.402)
KPS at Time of First Surgery (≤70/>70)	7/23	6/24	χ^2^ = 0.098; df = 1; *p* = 0.754 ^$^	Phi = −0.04 (−0.299–0.211)
GCS at Time of First Surgery (≤13/>13)	2/28	2/28	*p* = 1 ^π^	Phi = 0 (−0.245–0.251)
ASA Score at First Surgery	1 (1–3)	2 (1–3)	z(60) = 0.778; *p* = 0.437 ^#^	d = −0.10 (−0.608–0.405)
Hypertension (With/Without)	11/19	7/23	χ^2^ = 1.27; df = 1; *p* = 0.260 ^$^	Phi = −0.15 (−0.377–0.116)
DM (With/Without)	4/26	8/22	χ^2^ = 1.667; df = 1; *p* = 0.197 ^$^	Phi = 0.17 (−0.084–0.413)
Left Hemisphere (Yes/No)	19/11	17/13	χ^2^ = 0.278; df = 1; *p* = 0.598 ^$^	Phi = −0.07 (−0.316–0.189)
Eloquent Location Involvement (Motor/Sensory/Speech) (Yes/No)	18/12	18/12	χ^2^ = 0; df = 1; *p* = 1 ^$^	Phi = 0 (−0.268–0.245)
Completed Stupp Regimen (Yes/No)	30/0	27/3	*p* = 0.237 ^π^	Phi = −0.23 (−0.365–−0.122)
TERT Promoter Status (Mutated/Wildtype)	7/20	10/15	χ^2^ = 1.169; df = 1; *p* = 0.280 ^$^	Phi = −0.15 (−0.402–0.128)
MGMT Promoter Methylation Status (Present/Absent)	13/14	11/14	χ^2^ = 0.09; df = 1; *p* = 0.764 ^$^	Phi = −0.04 (−0.315–0.234)
GCS at Time Repeat Resection Offered (≤13/>13)	5/25	5/25	χ^2^ = 0; df = 1; *p* = 1 ^$^	Phi = 0 (−0.245–0.268)
KPS at Time Repeat Resection Offered (≤70/>70)	10/20	6/24	χ^2^ = 1.364; df = 1; *p* = 0.243 ^$^	Phi = −0.15 (−0.394–0.131)
Time to First Progression Since First Surgery (RANO 2.0) (Months)	5.6 (3–8)	5.05 (0–9.8)	z(36) = −0.745; *p* = 0.481 ^π^	d = 0.21 (−0.4496–0.861)
2nd-Line Chemotherapy at First Progression (Yes/No)	10/20	11/19	χ^2^ = 0.073; df = 1; *p* = 0.787 ^$^	Phi = −0.04 (−0.269–0.238)
Time to Offer Repeat Resection (months)	7.17 (1.9–14.5)	11.58 (3.8–57.07)	z(60) = 2.706; *p* = 0.007 ^#^	d = −0.84 (−13.601–−0.304)

**Table 2:** Survival and EOR in the repeat resection group (^#^ Mann–Whitney U; *p* < 0.05).

	One Surgery (*n* = 30)	Repeat Resection (*n* = 30)	*p*	Effect Size (95% CI)
Survival Time (Months)	9.17 (1.93–27.3)	23.87 (9.47–81.97)	z(60) = 5.108; *p* < 0.001 ^#^	Cohen’s d = −1.36 (−19.193–−0.793)
Survival Time After Repeat Resection Offered (Months)	2 (0.03–14.27)	11.35 (1.97–50.57)	z(60) = 4.348; *p* < 0.001 ^#^	Cohen’s d = −1.05 (−1.61–−0.467)
EOR at Repeat Resection (%)	-	88.17 (10.73–100)		
Residual Volume After Repeat Resection (cm^3^)	-	3.27 (0–94.84)		
RANO Class 1 Resection (Criteria Met/Unmet)		8/22		

**Table 3:** Comparison of short-term and long-term survivors in the repeat resection cohort (* Student’s *t*-test, ^#^ Mann–Whitney U, ^π^ Fisher’s Exact test, *p* < 0.05) (** MSM: motor, speech, and middle cerebral artery territory, Park score [11]).

Repeat Resection Cohort	Short-Term Survivors After 2nd Surgery (*n* = 7)	Long-Term Survivors After 2nd Surgery (*n* = 23)	Test Statistics, *p*	Effect Size (95% CI)
Survival after Repeat Resection (mo.s)	2.1 (2–3.5)	12.77 (6.1–50.6)	z(30) = 3.948; *p* < 0.001 ^#^	d = 14.41 (−2.356–−0.505)
Clinical Markers				
Age (Years)	50.57 (10.34)	48.96 (11.06)	t = 0.343; df = 28; *p* = 0.734 *	d = 0.15 (−0.700–0.994)
Gender (Female/Male)	3/4	18/5	*p* = 0.153 ^π^	Phi = −0.327 (−0.693–0.119)
GCS at Time of Repeat Resection (≤13/>13)	5/2	0/23	*p* < 0.001 ^π^	Phi = −0.811 (−1–−0.535)
Park Score	2 (1–3)	1 (0–2)	z(30) = −1.274; *p* = 0.203 ^#^	d = 0.53 (−0.382−1.41)
Preoperative Tumor Volume > 50 cm^3^	2/5	7/16	*p* = 1 ^π^	Phi = 0.017 (−0.365–0.343)
MSM **	3/4	11/12	*p* = 1 ^π^	Phi = 0.042 (−0.333–0.394)
KPS at Time of Repeat Resection (≤70/>70)	7/0	9/14	*p* = 0.007 ^π^	Phi = −0.516 (−0.725–−0.320)
Hypertension (With/Without)	3/4	4/19	*p* = 0.306 ^π^	Phi = −0.255 (−0.666–0.184)
DM (With/Without)	1/6	7/16	*p* = 0.638 ^π^	Phi = 0.154 (−0.189–0.426)
Surgical Markers				
Eloquent Location Involvement (Motor/Sensory/Speech) (Yes/No)	3/4	15/8	*p* = 0.392 ^π^	Phi = 0.193 (−0.198–0.558)
Left Hemisphere (Yes/No)	4/3	13/10	*p* = 1 ^π^	Phi = −0.005 (−0.367–0.373)
Touch to Ventricle Wall (Yes/No)	6/1	16/7	*p* = 0.638 ^π^	Phi = −0.154 (−0.426–0.177)
Ependymal Enhancement (Yes/No)	2/5	11/12	*p* = 0.427 ^π^	Phi = 0.164 (−0.200–0.484)
Preoperative Volume at Repeat Resection (cm^3^)	39.76 (5.61–67.24)	30.89 (3.47–145.9)	z(30) = −0.270; *p* = 0.811 ^#^	d = 0.13 (−0.979–0.715)
Postoperative Volume at Repeat Resection (cm^3^)	8.35 (0.62–25.16)	2.79 (0–94.84)	z(30) = −1.015; *p* = 0.335 ^#^	d = 0.19 (−1.035–0.660)
EOR at Repeat Resection (%)	78.99 (41.88–90.64)	89.45 (10.73–100)	z(30) = 1.163; *p* = 0.287 ^#^	d = 0.07 (−0.915–0.777)
RANO Class 1 at Repeat Resection (Criteria Met/Unmet)	0/7	8/15	*p* = 0.143 ^π^	Phi = 0.333 (0.184–0.498)
Pathological Markers				
MGMT Promoter Methylation Status (Present/Absent)	3/4	8/10	*p* = 1 ^π^	Phi = 0.014 (−0.379–0.385)
TERT Promoter Status (Mutated/Wildtype)	1/6	9/9	*p* = 0.179 ^π^	Phi = −0.327 (−0.612–0.053)

**Table 4:** Regression analysis of preoperative and postoperative tumor volumes at repeat resection. Model 1 uses postoperative volume (cm^3^) at repeat resection as the dependent variable (*n* = 30; s = 16.675; R^2^ = 0.433 (F = 21.371, *p* < 0.001); Shapiro–Wilk *p* = 0.041; Breusch–Pagan χ^2^(1) = 13.183, *p* < 0.001; Durbin–Watson test statistic = 1.734). Model 2 uses log-transformed postoperative volume at repeat resections as the dependent variable (*n* = 28 (2 patients were excluded); s = 0.438; R^2^ = 0.494 (F = 25.339, *p* < 0.001); Shapiro–Wilk *p* = 0.513; Breusch–Pagan χ^2^(1) = 38.186, *p* < 0.001; Durbin–Watson test statistic = 2.176).

Variable	B (95% CI)	Beta	t	*p*
Model 1: Constant	−2.393 (−11.884–7.097)	–	−0.517	0.610
Model 1: Preoperative Volume at Repeat Resection (cm^3^)	0.410 (0.228–0.592)	0.658	4.623	<0.001
Model 2: Constant	0.136 (−0.156–0.429)	–	0.957	0.347
Model 2: Preoperative Volume at Repeat Resection (cm^3^)	0.018 (0.011–0.025)	0.703	5.034	<0.001

**Table 5:** Regression analysis of postoperative residual tumor volume predicting survival after repeat resection. Model equation: Survival after Repeat Resection = 14.931 − 0.098 × Residual Volume (*n* = 30; s = 11.6829; R^2^ = 0.033 (F = 0.965, *p* = 0.334); Shapiro–Wilk *p* < 0.001; Breusch–Pagan χ^2^(1) = 3.376, *p* = 0.066; Durbin–Watson test statistic = 0.798).

Variable	B (95% CI)	Beta	t	*p*
Constant	14.931 (9.737–20.125)	–	5.888	<0.001
Postoperative Residual Volume (cm^3^)	−0.098 (−0.302–0.106)	−0.182	−0.982	0.334
Variable	B (95% CI)	Beta	t	*p*
Constant	14.931 (9.737–20.125)	–	5.888	<0.001
Residual Volume (cm^3^)	−0.098 (−0.302–0.106)	−0.182	−0.982	0.334
Variable	B (95% CI)	Beta	t	*p*
Constant	14.931 (9.737–20.125)	–	5.888	<0.001
Residual Volume (cm^3^)	−0.098 (−0.302–0.106)	−0.182	−0.982	0.334

## Data Availability

All the patient data generated and analyzed during the course of this study are available upon reasonable request from the corresponding author. Requests for data access can be directed to MM at mm2ee@virginia.edu. Data sharing will comply with institutional and journal policies, ensuring patient confidentiality and data security. Alternatively, other authors involved in this study may assist in facilitating access through appropriate channels.

## Supplementary Materials

The following supporting information can be downloaded at: https://www.mdpi.com/article/10.3390/brainsci15050463/s1, Supplementary File: Results of the descriptive and explanatory analysis of the patients.

## Abbreviations

The following abbreviations are used in this manuscript:

EOR	Extent of resection
GBM	Glioblastoma
rGBM	Recurrent GBM
MGMT	Methylguanine-DNA methyltransferase
ASA	The American Society of Anesthesiologists score
CE	Contrast enhancing
n-CE	Non-contrast enhancing

## References

[1] Stupp R., Mason W.P., Van den Bent M.J., Weller M., Fisher B., Taphoorn M.J.B., Belanger K., Brandes A.A., Marosi C., Bogdahn U. (2005). Radiotherapy plus Concomitant and Adjuvant Temozolomide for Glioblastoma. N. Engl. J. Med..

[2] Wen P.Y., Weller M., Lee E.Q., Alexander B.M., Barnholtz-Sloan J.S., Barthel F.P., Batchelor T.T., Bindra R.S., Chang S.M., Chiocca E.A. (2020). Glioblastoma in adults: A society for neuro-oncology (sno) and european society of neuro-oncology (eano) consensus review on current management and future directions. Neuro-Oncol..

[3] Tsien C.I., Pugh S.L., Dicker A.P., Raizer J.J., Matuszak M.M., Lallana E.C., Huang J., Algan O., Deb N., Portelance L. (2022). Nrg oncology/rtog1205: A randomized phase ii trial of concurrent bevacizumab and reirradiation versus bevacizumab alone as treatment for recurrent glioblastoma. J. Clin. Oncol..

[4] Ringel F., Pape H., Sabel M., Krex D., Bock H.C., Misch M. (2016). Clinical benefit from resection of recurrent glioblastomas: Results of a multicenter study including 503 patients with recurrent glioblastomas undergoing surgical resection. Neuro-Oncol..

[5] Karschnia P., Young J.S., Dono A., Häni L., Sciortino T., Bruno F., Juenger S.T., Teske N., Morshed R.A., Haddad A.F. (2022). Prognostic validation of a new classification system for extent of resection in glioblastoma: A report of the RANO resect group. Neuro-Oncol..

[6] Dono A., Zhu P., Holmes E., Takayasu T., Zhu J.J., Blanco A.I., Hsu S., Bhattacharjee M.B., Ballester L.Y., Kim D.H. (2022). Impacts of genotypic variants on survival following reoperation for recurrent glioblastoma. J. Neuro-Oncol..

[7] Whitfield B.T., Huse J.T. (2022). Classification of adult-type diffuse gliomas: Impact of the World Health Organization 2021 update. Brain Pathol..

[8] Hendrix J.M., Garmon E.H. (2025). American Society of Anesthesiologists Physical Status Classification System. StatPearls.

[9] Karschnia P., Dono A., Young J.S., Juenger S.T., Teske N., Häni L., Sciortino T., Mau C.Y., Bruno F., Nunez L. (2023). Prognostic evaluation of re-resection for recurrent glioblastoma using the novel RANO classification for extent of resection: A report of the RANO resect group. Neuro-Oncol..

[10] Wen P.Y., Bent M.v.D., Youssef G., Cloughesy T.F., Ellingson B.M., Weller M., Galanis E., Barboriak D.P., de Groot J., Gilbert M.R. (2023). RANO 2.0: Update to the Response Assessment in Neuro-Oncology Criteria for High- and Low-Grade Gliomas in Adults. J. Clin. Oncol..

[11] Park J.K., Hodges T., Arko L., Shen M., Dello Iacono D., McNabb A., Olsen Bailey N., Kreisl T.N., Iwamoto F.M., Sul J. (2010). Scale to predict survival after surgery for recurrent glioblastoma multiforme. J. Clin. Oncol..

[12] Gorlia T., Stupp R., Brandes A.A., Rampling R.R., Fumoleau P., Dittrich C., Campone M.M., Twelves C.C., Raymond E., Hegi M.E. (2012). New prognostic factors and calculators for outcome prediction in patients with recurrent glioblastoma: A pooled analysis of eortc brain tumour group phase i and ii clinical trials. Eur. J. Cancer.

[13] Wick W., Gorlia T., Bendszus M., Taphoorn M., Sahm F., Harting I., Brandes A.A., Taal W., Domont J., Idbaih A. (2017). Lomustine and bevacizumab in progressive glioblastoma. N. Engl. J. Med..

[14] Suchorska B., Weller M., Tabatabai G., Senft C., Hau P., Sabel M.C., Herrlinger U., Ketter R., Schlegel U., Marosi C. (2016). Complete resection of contrast-enhancing tumor volume is associated with improved survival in recurrent glioblastoma—Results from the DIRECTOR trial. Neuro-Oncol..

[15] Behling F., Rang J., Dangel E., Noell S., Renovanz M., Mäurer I., Schittenhelm J., Bender B., Paulsen F., Brendel B. (2022). Complete and incomplete resection for progressive glioblastoma prolongs post-progression survival. Front. Oncol..

[16] González V., Brell M., Fuster J., Moratinos L., Alegre D., López S., Ibáñez J. (2022). Analyzing the role of reoperation in recurrent glioblastoma: A 15-year retrospective study in a single institution. World J. Surg. Oncol..

[17] Tully P.A., Gogos A.J., Love C., Liew D., Drummond K.J., Morokoff A.P. (2016). Reoperation for Recurrent Glioblastoma and Its Association With Survival Benefit. Neurosurgery.

[18] Yong R.L., Wu T., Mihatov N., Shen M.J., Brown M.A., Zaghloul K.A., Park G.E., Park J.K. (2014). Residual tumor volume and patient survival following reoperation for recurrent glioblastoma. J. Neurosurgery.

[19] Molinaro A.M., Hervey-Jumper S., Morshed R.A., Young J., Han S.J., Chunduru P., Zhang Y., Phillips J.J., Shai A., Lafontaine M. (2020). Association of maximal extent of resection of contrast-enhanced and non-contrast-enhanced tumor with survival within molecular subgroups of patients with newly diagnosed glioblastoma. JAMA Oncol..

[20] Bjorland L.S., Mahesparan R., Fluge Ø., Gilje B., Kurz K.D., Farbu E. (2023). Impact of extent of resection on outcome from glioblastoma using the RANO resect group classification system: A retrospective, population-based cohort study. Neuro-Oncol. Adv..

[21] Louis D.N., Perry A., Wesseling P., Brat D.J., Cree I.A., Figarella-Branger D., Hawkins C., Ng H.K., Pfister S.M., Reifenberger G. (2021). The 2021 who classification of tumors of the central nervous system: A summary. Neuro-Oncol..

[22] Chen M.W., Morsy A.A., Liang S., Ng W.H. (2015). Re-do Craniotomy for Recurrent Grade IV Glioblastomas: Impact and Outcomes from the National Neuroscience Institute Singapore. World Neurosurg..

[23] Park Y.W., Choi K.S., Foltyn-Dumitru M., Brugnara G., Banan R., Kim S., Han K., Park J.E., Kessler T., Bendszus M. (2024). Incorporating Supramaximal Resection into Survival Stratification of IDH-wildtype Glioblastoma: A Refined Multi-institutional Recursive Partitioning Analysis. Clin. Cancer Res..

[24] Askun M.M., Zengin Y., Azizova A., Karli-Oguz K., Saydam O., Strobel T., Soylemezoglu F. (2024). Repeat Resection for Recurrent Glioblastoma in the WHO 2021 Era: A Prospective Matched Case-Control Study. Neurosurgery.

