# Supplementary material for: Repeat Resection for Recurrent Glioblastoma in the WHO 2021 Era: A Longitudinal Matched Case-Control Study
Source: Brain Sci. 2025 Apr 27;15(5):463. doi: 10.3390/brainsci15050463 (PMC12109614; doi:10.3390/brainsci15050463)
Supplement: Supplementary file 1 [file brainsci-15-00463-s001.zip › brainsci-3536764-supplementary.pdf]

SUPPLEMENTARY FILE: DETAILED RESULTS OF THE FACTOR ANALYSIS OF MIXED TYPE DATA TO IDENTIFY ANY OUTLIERS IN THE DATASET

Factor Analysis of Mixed Type Data (FAMD) results for baseline characteristics and clinical variables to identify outliers

Using age, preoperative tumor volume (cm3), gender, KPS, Eloquent as the variables below scree plot was obtained. It is seen that 4 dimensions provide explained variance of 86.2%.

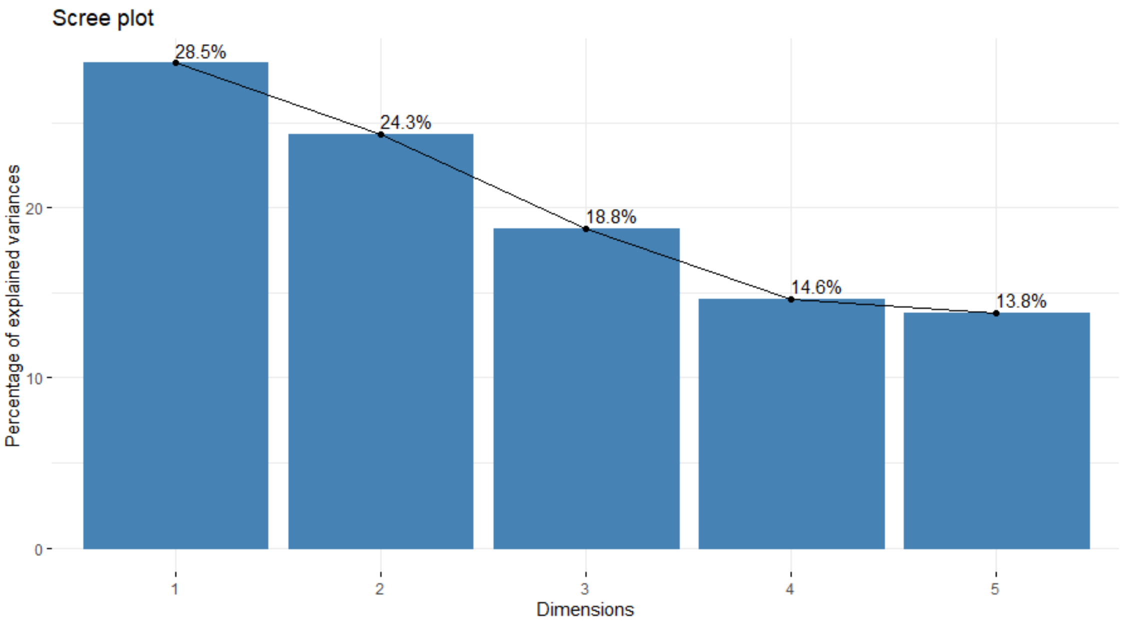

Figure S1. Scree Plot obtained from FAMD

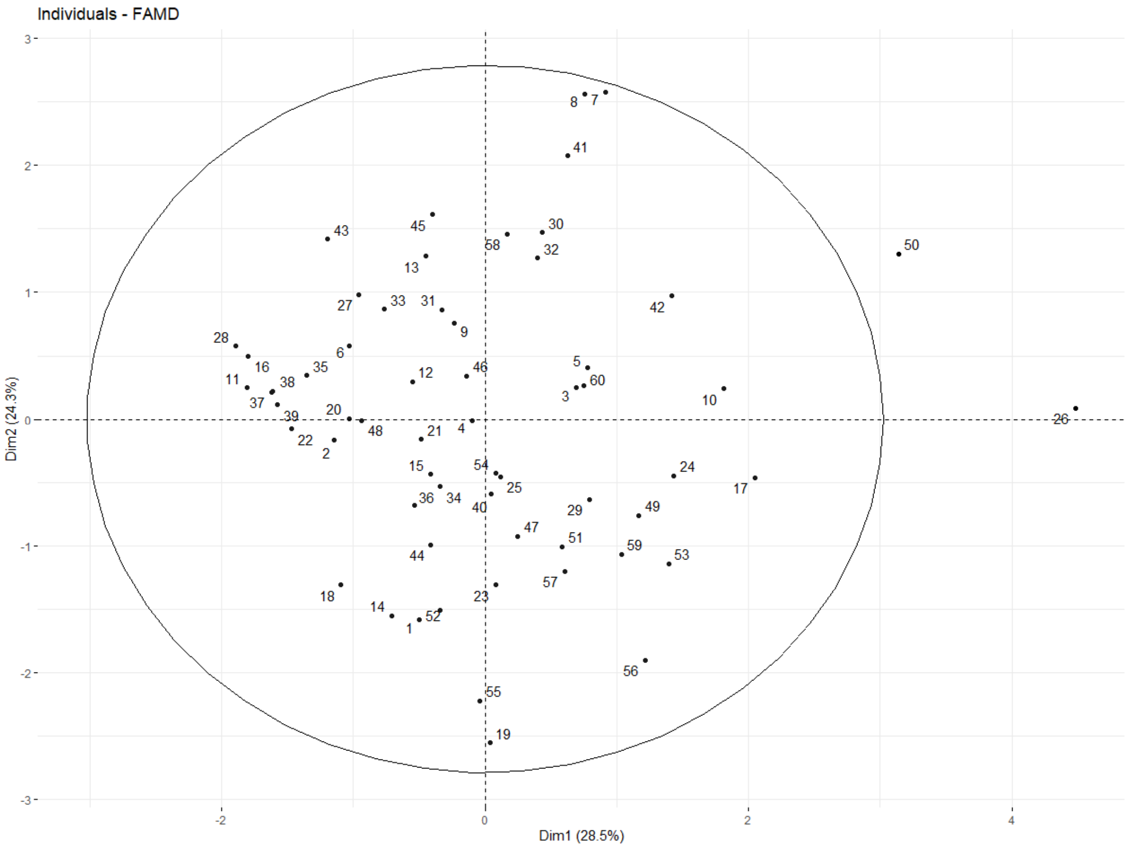

Figure S2. Scatter Plot of First and Second Dimensions

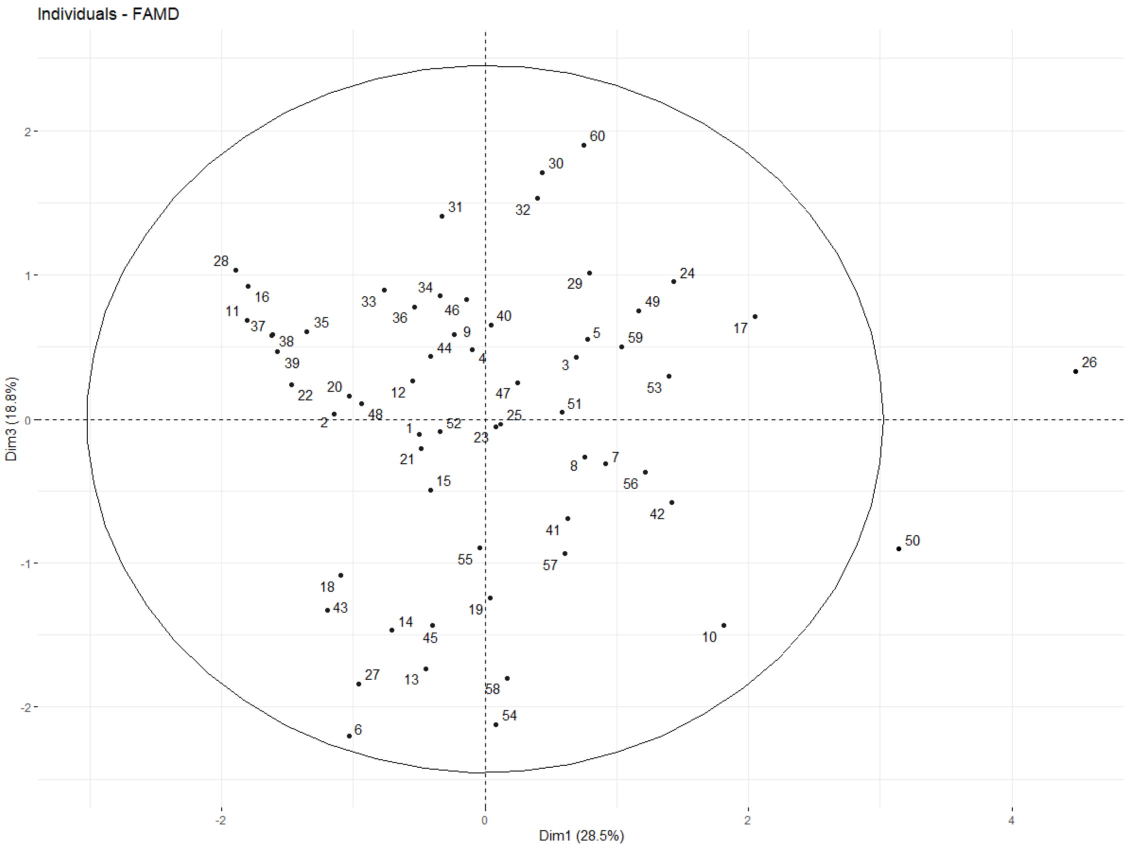

Figure S3. Scatter Plot of First and Third Dimensions

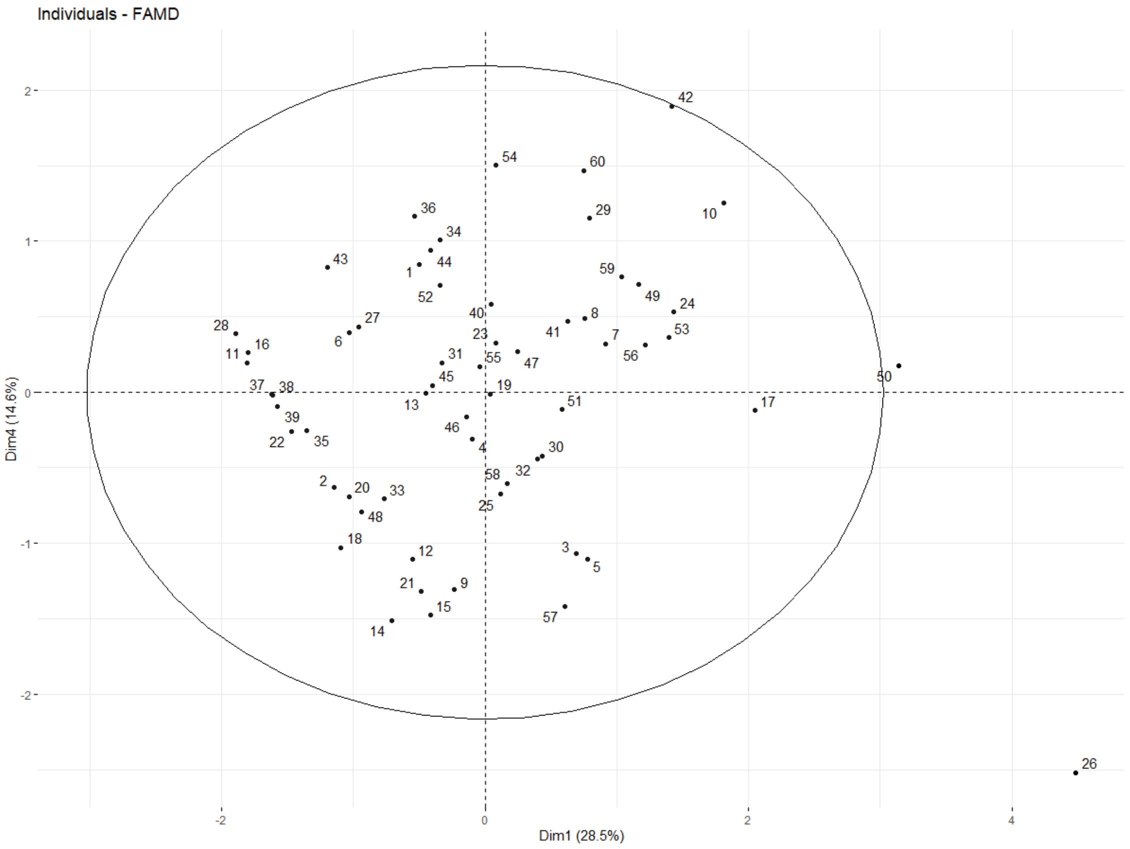

Figure S4. Scatter Plot of First and Fourth Dimensions

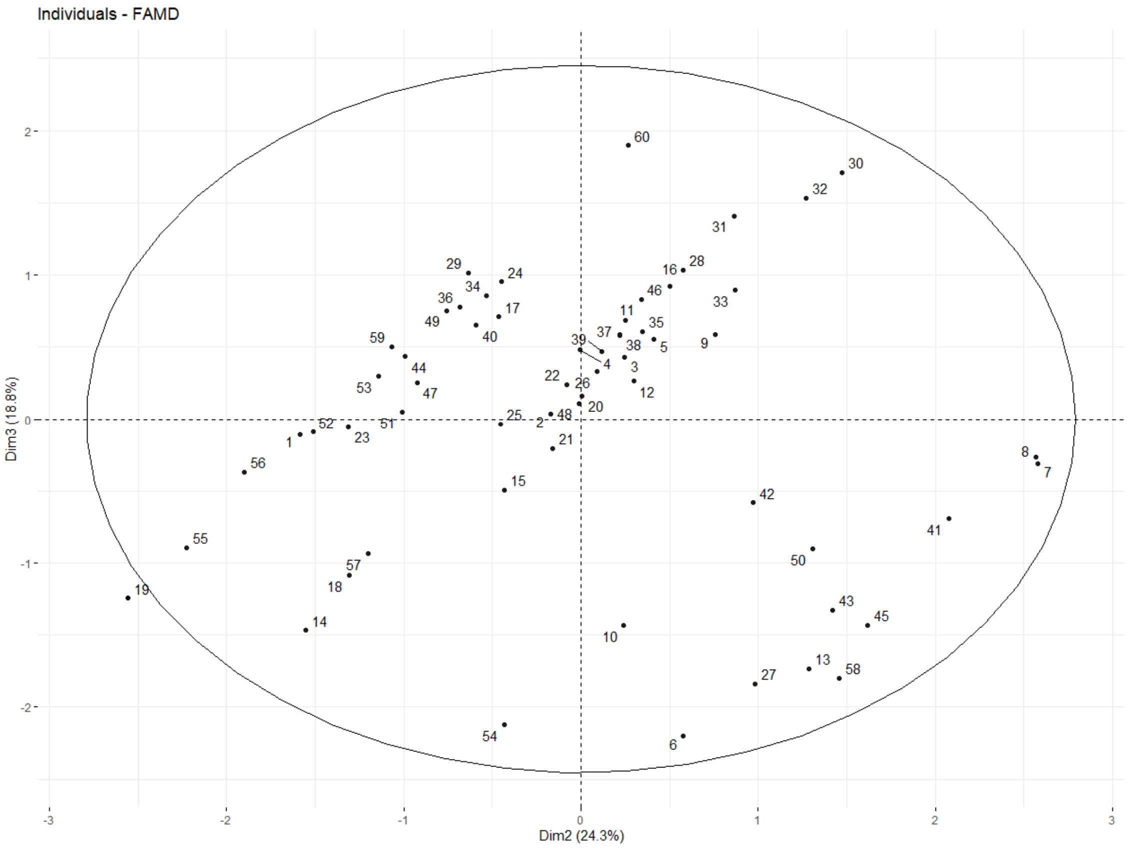

Figure S5. Scatter Plot of Second and Third Dimensions

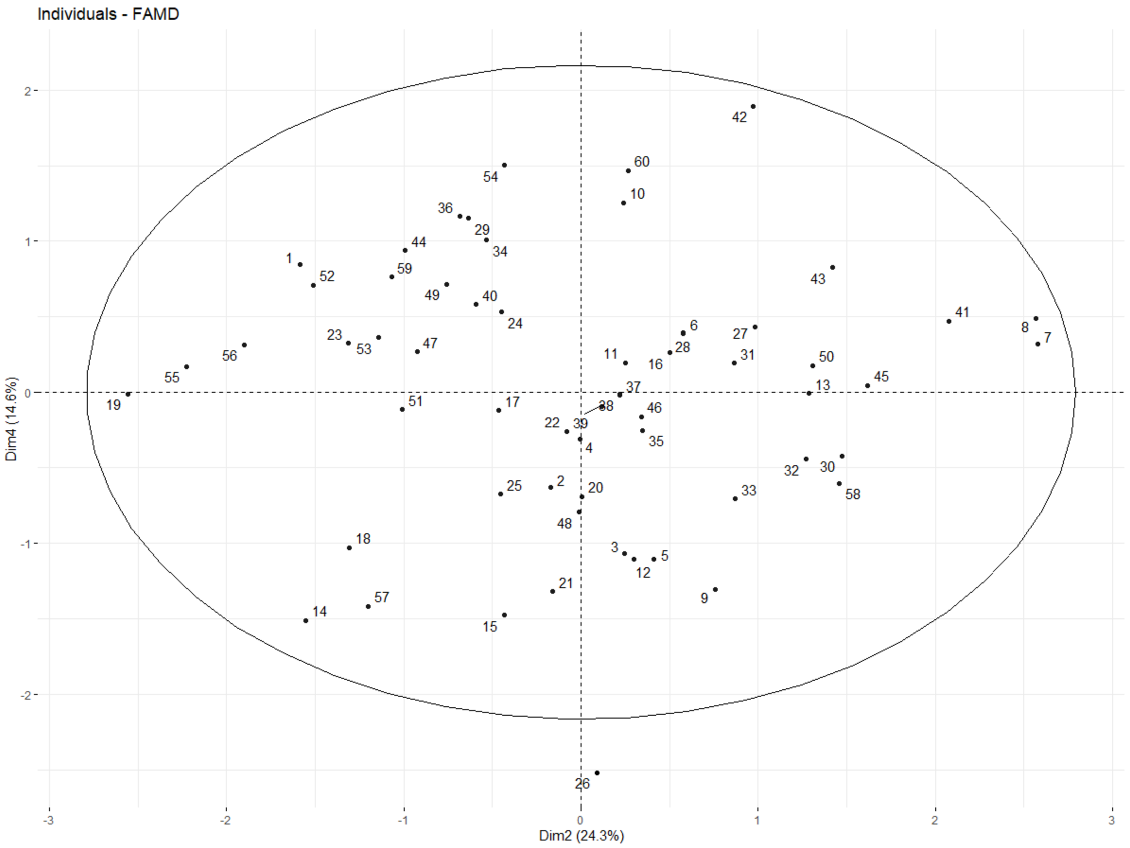

Figure S6. Scatter Plot of Second and Fourth Dimensions

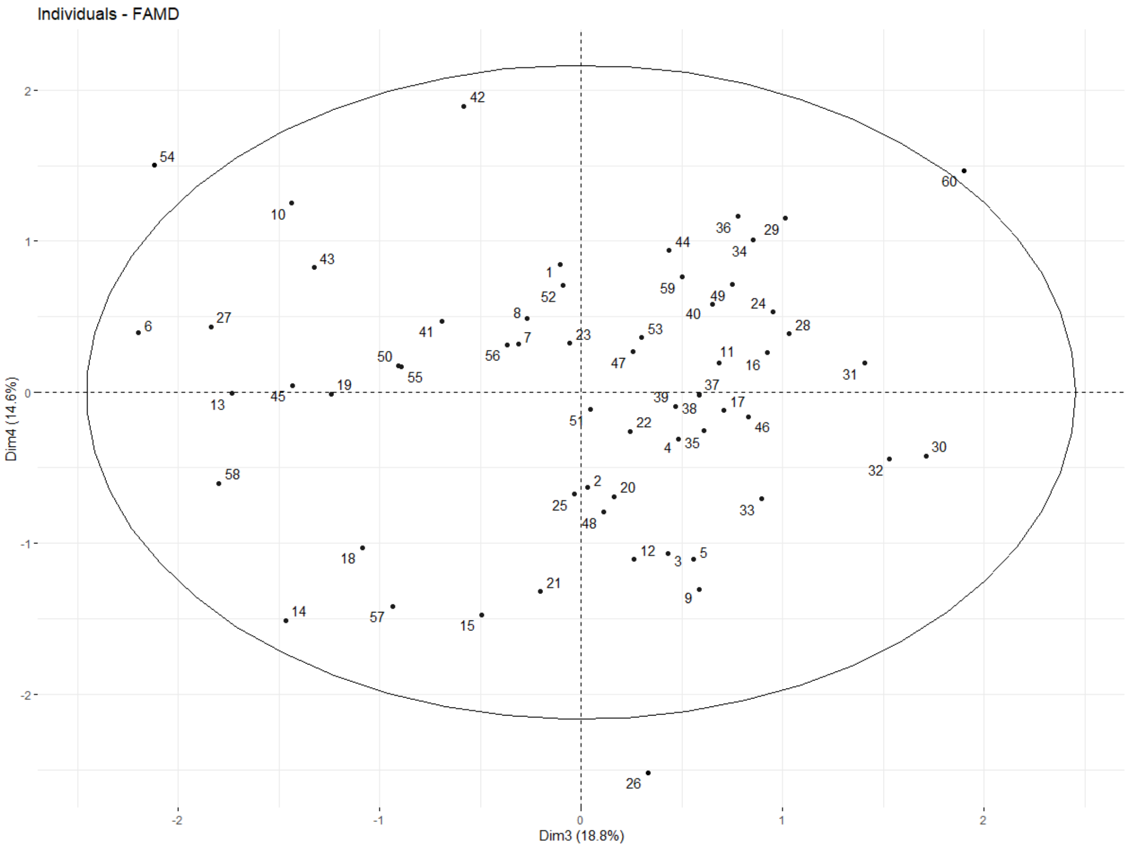

Figure S7. Scatter Plot of Third and Fourth Dimensions

Table S1. Correlation matrix of original variables and dimensions obtained from FAMD

Correlation Matrix of Variables with Components from Multiple Factor Analysis

	Dim.1	Dim.2	Dim.3	Dim.4	Dim.5
Age	0.06	0.4	0.37	0.12	0.05
Preop Tumor Volume	0.5	0.1	0	0.21	0.2
Gender	0.31	0.31	0	0.28	0.09
Baseline KPS	0.03	0.37	0.48	0.12	0
Eloquent Area	0.53	0.04	0.08	0.01	0.35

Based on the FAMD analysis, observations numbered 26, 50, 54 and 60 are identified as potential outliers, as indicated by the plots of principal component pairs, which visually highlight deviations from the general pattern in the data. However, these observations are not excluded from the analysis due to their clinical importance.

We have performed a **FAMD** (Factor Analysis of Mixed Data) approach, which is suitable for our mixed dataset (categorical and continuous variables). A **scree plot** illustrating the variance captured by each principal component has been added. We have also visualized all participants in pairwise principal component planes (e.g., PC1 vs. PC2, PC3 vs. PC4, etc.) to evaluate potential outliers, and **observations 26, 50, 54, and 60** emerged as possible outliers based on their deviation from the overall data pattern.

Nevertheless, we retained these cases due to their clinical significance. Additionally, we included a table demonstrating **the correlations between original variables and the extracted principal components**, exploring any coefficients greater than ±0.3 to identify key associations.
